# Longitudinal assessment of injury recidivism among adults in the United States: findings from a population-based sample

**DOI:** 10.1186/s40621-016-0071-x

**Published:** 2016-02-02

**Authors:** Suliman Alghnam, Glen H. Tinkoff, Renan Castillo

**Affiliations:** 1 King Abdullah International Medical Research Center, King Saud Bin Abdulaziz University for Health Sciences, KAIMRC, KSAU-HS, Riyadh, Saudi Arabia; 2 Department of Health Policy and Management, Johns Hopkins Bloomberg School of Public Health, HH 598 624 N. Broadway, Baltimore, MD 21205 USA; 3 Department of Surgery, Christiana Care Health System, Newark, DE 19718 USA

**Keywords:** Injury burden, Recidivism, Repeated injuries, MEPS

## Abstract

**Background:**

Repeated injuries, as known as injury recidivism, pose a significant burden on population health and healthcare settings. Therefore, identifying those at risk of recidivism can highlight targeted populations for primary prevention in order to improve health and reduce healthcare expenditures. There has been limited research on factors associated with recidivism in the U.S. Using a population-based sample, we aim to: 1) identify the prevalence and risk factors for injury recidivism among non-institutionalized adults; 2) investigate the trend in nationwide recidivism rates over time.

**Methods:**

Using the Medical Expenditure Panel Survey (MEPS), 19,134 adults with at least one reported injury were followed for about 2 years. Reported injuries were those associated with healthcare utilization, disability days or any effects on self-reported health. The independent associations between risk factors for recidivism were evaluated incorporating a weighted logistic regression model.

**Results:**

There were 4,136 recidivists representing over nine million individuals in the U.S. over a 2-year follow-up. About 44 % of recidivists sustained severe injuries requiring a hospitalization, a physician’s office visit or an emergency department visit. Compared with those who sustained a single injury, recidivists were more likely to be white, unmarried, reside in metropolitan areas, and report a higher prevalence of chronic conditions. Age, sex, race/ethnicity, marital status, urbanicity, region, diabetes, stroke, asthma and depression symptoms were significant predictors of recidivism. Significant interaction effects between age and gender suggested those in the 18–25 age group, the odds of being a recidivist were 1.45 higher among males than females adjusting for other covariates. While having positive screens for depression in both follow-up years was associated with 1.46 (95 % CI = 1.21–1.77) higher odds of recidivisms than the reference group adjusting for other variables.

**Conclusions:**

We observed a higher recidivism rate among injured individuals in this study than previously reported. Our findings emphasize the pressing need for injury prevention to reduce the burden of repeated injuries. Preventative efforts may benefit from focusing on males between 18 and 25 years of age and those with comorbidities such as diabetes, stroke and depression.

## Background

Over 30 million patients in the United States (U.S.) were treated in emergency departments (ED) due to injuries in 2013. (2013) Adding to this burden is the fact that injured patients remain at risk for injury reoccurrence, as known as injury recidivism (Caufeild et al. 2004; Kaufmann et al. 1998). Consequently, patients suffer further decline in health and healthcare settings incur high expenditures due to increase utilization. Therefore, identifying individuals at risk of recidivism can highlight targeted populations for “primary prevention” (Toschlog et al. 2007) in order to improve population health and reduce medical costs.

While previous literature has focused on identifying risk factors for sustaining injuries (Rasouli et al. 2011; Rivara et al. 2015), much remains unclear about factors associated with injury recidivism. One study (Toschlog et al. 2007) found females to be more likely than males to have recurrent injuries while the opposite was found by other studies (Caufeild et al. 2004; McCoy et al. 2013; Toschlog et al. 2007; Worrell et al. 2006). For the most part, data limitations hinder the ability to provide comprehensive evidence because most studies used registries collected at a single hospital (Davis et al. 2013; Dixon et al. 2014; Kondo et al. 2011; Kwan et al. 2011; McCoy et al. 2013). It is not unlikely that patients sustaining a recurrent injury may go to a different provider or choose not seek medical care either due to access factors, negative experience with providers or lower severity of later injuries. Consequently, recidivism rates in the literature vary with estimates between 1 % and 44 % (Caufeild et al. 2004). More comprehensive approaches such as the use of population-based surveys may overcome such hurdles in shedding more light on the burden of injury recidivism.

To the best of our knowledge, no previous study has used a population-based dataset to evaluate injury recidivism in the U.S. Furthermore, prior studies focused on injuries associated with hospitalizations, which may not reflect the true burden of injuries on population health. As such, the aims of this study are twofold: 1) to identify, using a population-based sample, the prevalence and risk factors for injury recidivism; 2) to investigate the trend in nationwide rates of recidivism over the period between years 2004 and 2010.

## Methods

### Dataset

This longitudinal study used the Medical Expenditure Panel Survey (MEPS), a household survey of U.S. non-institutionalized populations. The Agency for Healthcare Research and Quality (AHRQ) has administered and maintained the MEPS since 1996 (AHRQ 2006). This nationally representative dataset includes overlapping panels based on the previous year’s sample of the National Health interview Survey (NHIS). Every year, a new panel is recruited and followed for about 30 months. Throughout the follow-up period, participants are interviewed in their homes five times with a time lag of 4–5 months.

**Fig. 1:**
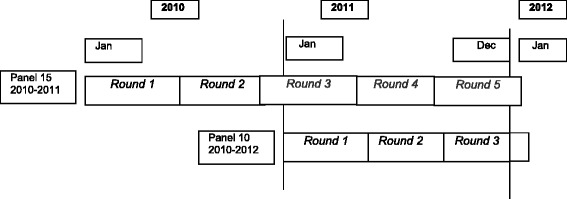
Survey administration in the Medical Expenditure

The MEPS collects information on variety of areas including demographics, health status, healthcare use and expenditures of all participants. To achieve the objectives of the present study, two files were used: the characteristic and the medical conditions files. Panels 9–16, which started between 2004 and 2010, were pooled for the purpose of the analysis. Each panel follows participants for about 2 years as shown on Fig. 1 (i.e. Panel 16 was initiated in 2010 and follow-up ended sometimes in mid 2012).

### Study population

This study included participants 18 years of age or older with at least one reported injury during the study period. When a respondent reports an injury, the interviewer also asks about the date of the incident. Because the focus of this analysis is on short-term recidivism, we limited our sample to injuries sustained either: in the year prior to recruitment or during the study follow-up.

### Outcome measure

The main dependent variable is injury recidivism during the follow-up period.

Reported injuries are defined as those associated with healthcare utilization (hospital stay, outpatient visit, ED visit, home health episode, prescribed medication purchase, or medical provider visit), the reason for one or more episodes of disability days or any condition “bothering” the person during the reference period (AHRQ 2006). In addition, we employed a more restrictive injury definition in order to examine severe injuries including: those associated with at least one hospitalization, a physician’s office visit or an ED visit. One of the survey questions asks about the round in which the injury was first reported. We used this variable to identify recidivists by determining if other injury-related incidents occurred later during the follow-up period.

Two main analyses were conducted. First, we evaluated the association between several variables and injury recidivism. Second, we evaluated the percentage of recidivists by panel to assess whether there are any trends throughout the study.

### Covariates

Some of the covariates considered for the analysis were: age in years (18–25, 26–45, 46–64 and ≥65), gender, income (as a percentage of poverty level [poor (<100 %), near poor (100– <125 %), low income (125– <200 %), middle (200– <400 %), and high income (≥400 %)], education (high school or below, some college, bachelors, and beyond bachelors), and race/ethnicity (white, blacks, Hispanic and others). The “other” race category included those reporting being Asians, American Indians, native Hawaiian and those reporting multiple races. Binary variables included an indicator for living in a metropolitan statistical area (MSA status), census region (Northwest, Midwest, South and West), an indicator for being a current smoker, and indicator for being married. The selection of these potential risk factors was based on prior literature (Alghnam et al. 2013; Kaufmann et al. 1998; Cochran et al. 2014; McCoy et al. 2013; Sayfan and Berlin 1997; Toschlog et al. 2007).

Depression is another risk factor for injury and potentially recidivism (Asbridge et al. 2014). To assess the association between depressive symptoms and recidivism, we used a variable based on the Patient Health Questionnaire (PHQ-2) score. This instrument is used to screen for depressive symptoms and was found to have high sensitivity and specificity (Godha et al. 2010). The PHQ-2 score ranges between 0 and 6 and a score of 3 or higher is considered a positive screen for depressive symptoms. Because the PHQ-2 was measured twice during follow-up, we created a categorical variable: negative depressive symptoms in both years, one positive screen for depression, and positive screens for depressive symptoms in both years.

Additionally, we considered a set of indicator variables for several health conditions that are known to affect health status. These included diabetes, asthma, emphysema, stroke, hypertension and coronary heart diseases. Those conditions are referred to in the MEPS as “priority conditions” (2003). Such classification was designated because they are relatively prevalent, affect health status, and generally accepted standards for appropriate care have been developed. Participants were asked if they had ever been diagnosed with the condition (yes or no).

### Alcohol use

One of the relevant factors for injury recidivism is alcohol use (Toschlog et al. 2007). In the area of motor vehicles crashes, for instance, alcohol use was found to be among the strongest predictors of injury and mortality (Sommers et al. 2006). Although the MEPS does not ask respondents about their alcohol intake during the in-person interview, the medical conditions file contains information on alcohol-related health conditions. This may allow investigating the association between alcohol and injury recidivism. Reported medical conditions, based on the criteria listed above, were also used to create a variable for alcohol use among the study participants. After participants report health conditions to the interviewer during the survey, trained personnel from AHRQ convert those conditions into ICD-9 codes. Codes are then reduced to the first three digits for confidentiality purposes. Next, similar ICD-9 codes are grouped into 261 unique clinical classification codes (CCCs), which represent groupings of clinically similar medical conditions (Mukherjee et al. 2011). Participants were categorized as having an alcohol-related condition if any of the following codes was documented: alcohol dependence syndrome (ICD9 = 303), alcohol-related disorders (ICD9 = 660), alcohol toxicity (ICD9 = 980), alcohol-related mental disorders (CCC = 66), or alcohol-related liver diseases (CCC = 150).

### Statistical analysis

The MEPS survey has a complex survey design, which takes into account survey weights, strata and clustering of individuals in order to provide nationally representative results of U.S. non-institutionalized populations. Therefore, all analyses, including standard errors, were adjusted for the design using STATA 14 (STATA Corp LP, College Station, Tex) and its survey procedures (Heeringa and West 2010). Descriptive characteristics by recidivism status were obtained. Bivariate analyses were explored using Chi-2 tests and a *p*-value of *p* < 0.05 was considered statistically significant. These analyses were also performed looking only at severe injuries as defined previously.

Weighted logistic regression models were constructed with the binary outcome recidivism (0 = no recidivism, 1 = recidivism) adjusting for potential confounders. This was carried out for both any injury recidivism and severe injuries recidivism. Initially, all potential variables were considered in the models regardless of significance in the bivariate analysis. However, only those significant (*p*-value <0.05) were included in the final model with the exception of age and sex, which were forced into the models. An overall interaction term between age and sex was significant (*p* < 0.01) and thus was included in the analyses. Other interactions between sex by metropolitan area, race by education and race by metropolitan area were tested, but were not significant. The results are presented as odds ratios (OR) with 95 % confidence intervals. To interpret interaction coefficients in the model, the STATA command *lincom* was utilized to calculate the odds ratios between specific groups and their associated confidence intervals. Variables included in the model investigating injury recidivism were: age, sex, race/ethnicity, marital status, MSA status, region, diabetes, asthma, stroke and depression while variables included in the model investigating severe injury recidivism were: age, sex, race/ethnicity, region, insurance, asthma, depression and hypertension.

To examine the change in recidivism rates throughout the study period, we plotted the proportion of recidivists by the study panels 9–16. Excel Microsoft 2011 for Mac was used to produce the plot. To explore if there was any significant change in recidivism across follow-up years, we performed a trend test (Sribney 2015). This was also performed for a subgroup of “severe” injuries defined as injuries associated with at least a hospital stay, an ED visit, or a physician’s office visit. Because this study used publically available de-identified data, it was granted an exempt status by the Johns Hopkins School of Public Health Institutional Review Board.

## Results

### Descriptive characteristics: any injury

During the study period, 141,054 individuals were recruited as MEPS participants in panels 9–16. Of those, 19,134 (13.5 %) individuals met the study inclusion criteria and were included in the analysis. During the study, 4136 reported recurrent injuries. After taking sampling weights, clusters and strata into account, these data suggest that 17.9 % of those injured were recidivists. This represents over nine million adults in the U.S. who experience injury recidivism over a 2-year follow-up (Table 1).

**Table 1:** Descriptive characteristics of the injured population by re-injury status

Variable	Single injury weighted	Recidivist weighted	Total weighted	*P*-Value
*N* (un-weighted)	9,071,398 (4136)	41,911,119 (14,998)	50,982,517 (19,134)	
Age category (%)				0.95
18–24	13.1	13.1	13.1	
25–45	36.9	36.2	36.8	
46–64	32.5	33.2	32.6	
≥ 65	17.6	17.5	17.6	
Male (%) ^a^	49.5	50.9	49.8	0.16
Income (%)^a^ ^b^				0.31
Poor (<100 %)	11.7	12.5	11.8	
Near poor (100– <125 %)	4.2	4.3	4.2	
Low (125– <200 %)	13.5	14.4	13.6	
Middle (200– <400 %)	31.4	30.8	31.3	
High (≥400 %)	39.2	38.1	39.0	
Race/ethnicity (%)^a^				<0.01
White	75.1	78.4	75.7	
Black	9.5	8.7	9.4	
Hispanic	10.2	8.1	9.8	
Other	5.2	4.8	5.1	
Highest degree (%)^a^				0.47
High school or below	16.3	16.4	16.3	
Some college	31.0	29.7	30.8	
Bachelor	25.7	27.4	26.0	
Beyond	27.0	26.5	26.9	
Insurance status (%)^a^				0.09
Private	70.4	69.7	70.3	
Public	16.3	17.5	16.5	
Uninsured	13.4	12.8	13.3	
In a metropolitan area (%)^a^	82.5	84.6	82.8	0.02
Region				<0.01
Northeast	18.2	17.8	18.1	
Midwest	24.3	26.1	24.6	
South	34.1	29.2	33.2	
West	23.4	27.0	24.1	
Married (%)^a^	49.5	44.3	48.6	<0.01
Diabetes (%)^a^	8.6	10.9	9.0	<0.01
Coronary heart disease (%)^a^	5.1	5.9	5.3	0.26
Asthma (%)^a^	11.6	15.2	12.3	<0.01
Stroke (%)^a^	4.1	5.4	4.3	<0.01
Emphysema (%)^a^	2.1	2.2	2.1	0.92
Hypertension (%)^a^	31.8	32.8	32.0	0.35
Alcohol-related health conditions (%)^a^	0.8	0.8	0.8	0.89
Depression screener positive				<0.01
Neither years	82.4	78.8	81.7	
One year	12.7	14.1	12.9	
Both years (%)^a^	4.8	7.1	5.2	
Smoking (%)^a^	22.3	25.3	22.8	<0.01

^a^Age-adjusted (2000 U.S. population)

^b^Percentage of poverty line

Compared with those who sustained a single injury, recidivists were more likely to be white, unmarried, and reside in metropolitan areas. While recidivists were more likely than non-recidivists to report diabetes (10.9 vs. 8.6 %, *p* < 0.01), asthma (15.2 vs. 11.6 %, *p* < 0.01) and, smoking (25.3 vs. 22.3 %, *p* < 0.01), there were no difference in the frequency of coronary health diseases or alcohol related conditions between the two groups. Recidivist were also more likely to have a positive screen for depression than non-recidivist (*p* < 0.01).

### Descriptive characteristics: severe injuries

The results suggest that 12.4 % of those sustaining severe injuries were recidivists representing four million adults in the U.S. (Table 2). Recidivists were more likely to be positive for depressive symptoms, carry public insurance and to have higher prevalence of diabetes, asthma, stroke, and hypertension (*p* < 0.01).

**Table 2:** Descriptive characteristics of those who sustained severe injuries by recidivism status

Variable	Single injury weighted	Recidivist weighted	Total weighted	*P*-Value
*N* (un-weighted)	4,020,836 (1342)	28,279,731 (10,275)	32,300,576 (11,617)	
Age category (%)				0.21
18–24	13.0	11.1	12.7	
25–45	35.4	34.3	35.3	
46–64	33.0	33.7	33.1	
≥ 65	18.7	20.8	18.9	
Male (%)^a^	48.5	47.2	48.3	0.36
Income (%)^a^ ^b^				0.96
Poor (<100 %)	12.3	13.0	12.4	
Near poor (100– <125 %)	4.5	4.7	4.5	
Low (125– <200 %)	13.5	13.3	13.5	
Middle (200– <400 %)	31.2	31.1	31.2	
High (≥400 %)	38.5	37.9	38.4	
Race/ethnicity (%)^a^				<0.01
White	75.4	80.2	76.0	
Black	9.9	8.4	9.7	
Hispanic	9.8	7.3	9.5	
Other	4.9	4.1	4.8	
Highest degree (%)^a^				0.47
High school or below	16.9	14.8	16.7	
Some college	31.3	30.8	31.2	
Bachelor	25.9	27.6	26.1	
Beyond college	25.9	26.7	26.0	
Insurance status (%)^a^				<0.01
Private	70.1	69.6	70.1	
Public	17.6	21.8	18.1	
Uninsured	12.3	8.6	11.8	
In a metropolitan area (%)^a^	82.4	81.9	82.0	0.57
Region				<0.01
Northeast	18.6	20.7	18.9	
Midwest	24.9	28.0	25.3	
South	33.8	28.1	33.1	
West	22.7	23.2	22.8	
Married (%)^a^	49.1	47.3	48.9	0.36
Diabetes (%)^a^	9.4	12.0	9.7	0.01
Coronary heart disease (%)^a^	5.8	7.9	6.1	0.04
Asthma (%)^a^	12.2	16.3	12.7	<0.01
Stroke (%)^a^	4.6	6.6	4.9	<0.01
Emphysema (%)^a^	2.3	2.9	2.4	0.18
Hypertension (%)^a^	33.4	38.5	34.0	<0.01
Alcohol-related health conditions (%)^a^	0.8	0.7	0.7	0.61
Depression screener positive				<0.01
Neither years	81.2	77.4	80.7	
One year	13.4	15.0	13.6	
Both years (%)^a^	5.2	7.5	5.5	
Smoking (%)^a^	23.1	24.3	23.2	0.46

^a^Age-adjusted (2000 U.S. population)

^b^Percentage of poverty line

### Regression analyses

The regression model of any injury identified age, sex, race/ethnicity, marital status, diabetes, asthma, depressive symptoms, region, and MSA status to be independent predictors of injury recidivism. Having two positive screens for depression was associated with 1.46 (95 % CI = 1.21–1.77) higher odds of recidivisms than the reference group adjusting for other variables. Diabetics were 1.37 (95 % CI = 1.10–1.62) more likely to be recidivists than those injured without diabetes adjusting for covariates (Table 3). Similar odds were found looking at asthma and the risk of injury recidivism (OR = 1.28, 95 % CI = 1.13–1.44). Interactions effects between sex and age groups were statistically significant indicating higher likelihood of injury recidivism among younger males relative to females. Among those in the 18–25 age group, the odds of being a recidivist were 1.45 higher among males than females adjusting for other covariates. The opposite was true among those 65 and older, in which injured males were 27 % less likely to sustain recurrent injuries (Table 3).

**Table 3:** Adjusted odds ratios from the logistic regression analysis of predictors of injury recidivism

Variables	Any recidivism OR (95 % CI)	Severe injury recidivism OR (95 % CI)
	Comparing *males* to females by age category	
Age categorized [years]		
18–25	1.45 (1.06–1.99)	1.35 (0.87–2.09)
26–45	1.43 (1.21–1. 68)	1.41 (1.09–1.83)
46–64	0.92 (0.73–1.09)	0.80 (0.65–1.04)
65≥	0.73 (0.56–0.95)	0.71 (0.52–1.06)
Race/ethnicity		
White	Reference	Reference
Black	0.83 (0.72–0.95)	0.78 (0.64–0.95)
Hispanic	0.70 (0.58–0.81)	0.71 (0.55–0.92)
Other	0.88 (0.71–1.09)	0.79 (0.57–1.08)
Insurance status		
Private	NS	Reference
Public		1.31 (1.09–1.52)
Uninsured		0.74 (0.56–0.95)
In a metropolitan area	1.2 (1.06–1.36)	NS
Region		
Northeast	Reference	Reference
Midwest	1.14 (0.96–1.34)	1.07 (0.86–1.34)
South	0.90 (0.75–1.07)	0.77 (0.61–0.97)
West	1.21 (1.01–1.44)	0.96 (0.76–1.22)
Depression screener positive		
Neither years	Reference	Reference
One year	1.15 (1.0–1.32)	1.14 (0.93–1.42)
Both years	1.46 (1.21–1.77)	1.36 (1.05–1.77)
Asthma	1.28 (1.13–1.44)	1.30 (1.04–1.62)
Stroke	1.26 (1.01–1.60)	NS
Diabetes	1.37 (1.10–1.62)	NS
Hypertension	NS	1.23 (1.06–1.40)
Married	0.81 (0.74–0.88)	NS

*NS* not significant

Overall, similar findings were observed when looking at severe injury recidivism. However, hypertension and health insurance were significant predictors recidivism. Those with public insurance were 1.31 (95 % CI = 1.09–1.52) more likely than the privately insured to be recidivists. Having two positive screens for depressive symptoms was the strongest predictor among comorbidities with 1.36 higher odds of recidivism than those without depressive symptoms.

**Fig. 2:**
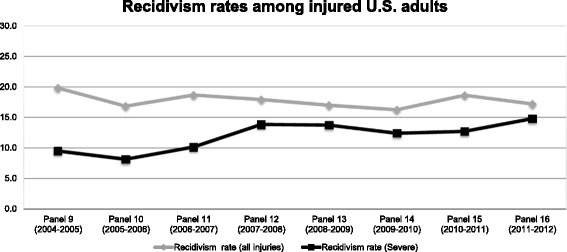
Recidivism rates among U.S. non-institutionalized populations across the study panels. *Severe: an injury that was associated with at least one hospitalization, emergency department visit or a physician’s office visit

The trend test suggested that there was no significant change in the proportion of recidivist throughout the study period (*p*-value = 0.18). This proportion ranged from 19.8 % in panel 9 to 17.2 % in panel 16 (Fig. 2). On the other hand, a significant increase (*p*-value <0.01) in recidivism rates was found when looking only at severe injuries with the proportion of recidivists increasing from 9.5 % in panel 9 to 14.8 % in panel 16.

## Discussion

This is the first study that examined injury recidivism in using a nationally representative sample. We found age, sex, race/ethnicity, marital status, urbanicity, region, diabetes, stroke, asthma and depression to be significant predictors of injury recidivism. Our finding that over nine million injured individuals suffer a recurrent injury is a strong evidence for the staggering implications of injuries on population health. This serves as a reminder of the importance of further investment in injury prevention programs.

Elevated depressive symptoms was among the strongest predictors of recidivisms in our study. Those with two positive screens for depression are likely to have chronic depression and were found at higher risk of recidivism than other groups despite adjusting for other health conditions. This finding is of particular interest because self-harm injury is a concern among this group and therefore, individuals with depression warrant carful assessment by treating clinicians in order to prevent further injuries (Nixon et al. 2008). A study that examined patients who committed suicide found that half of those who died had a previous admission to the same trauma center (Kiankhooy et al. 2009). Future studies should aim to understand specific injury mechanisms most common among recidivists with depression to better plan prevention programs.

Our findings suggest that younger age and male gender are associated with higher risk of injury recidivism, which is consistent with previous reports (Kaufmann et al. 1998; Dixon et al. 2014). Nevertheless, none of the previous studies investigated the interrelationship between sex and age in the risk of recidivism. We postulate that risk taking behavior associated with this group may explain the elevated risk of injury recidivism (Hefny et al. 2012; Irwin and Millstein 1986). Therefore, preventative efforts aimed at this group may help reduce the burden of recidivism on population health.

The recidivism rate of 17.9 % found in our study is higher than previously reported in other studies (Kaufmann et al. 1998; Davis et al. 2013; Kwan et al. 2011; Rivara et al. 1993). This discrepancy may be attributed to differences in methodology, catchment population or the timeframe of the study. Another major factor is the fact that most previous studies have relied on hospital admission data. In this study, we did not restrict our sample to injuries leading to healthcare utilization, thus, we had the advantage of capturing a wider spectrum of injury severity in addition to being more representative of the U.S. population.

We found those diagnosed with diabetes, stroke or asthma to be at increased risk of recidivism. Several studies have found diabetes to be associated with increased risk of injuries (Dede et al. 2014; Malmivaara et al. 1993; Yau et al. 2013). This increased risk of injuries may be attributed to microvascular and macrovascular complications due to the progression of diabetes (Mendes et al. 2013). A study by Malmivaara et al. examined predictors of injury and found those with diabetes had a higher risk of sustaining a fall-related injury than those without diabetes (Malmivaara et al. 1993). Similarly, those who suffered a stroke are more likely to sustain injuries, particularly due to falls. (Batchelor et al. 2012; Vu et al. 2011; Whitson et al. 2006) Furthermore, asthma has been found in both pediatric and adult populations to be an independent risk factor for sustaining injuries. (Garg and Silverberg 2014; Liang et al. 2011; Schwebel and Brezausek 2009) What our findings add to the literature is the fact that these conditions are also associated with injury recidivism. Nevertheless, more work is needed to better understand how to reduce recidivism risk among patients with those conditions.

Unlike overall injuries, we found a slight increase in severe injury recidivism throughout the study period. Surprisingly, this increase took place as the American College of Surgeons introduced in 2007 a mandate for alcohol Screening and Brief Intervention (SBI). This mandate aimed to reduce the consequences of alcohol abuse, one of which is injury recidivism (Terrell et al. 2008). Earlier studies have found SBI to be associated with reduction in alcohol intake and rates of injury recidivism (Gentilello et al. 1999; Rivara et al. 1993). Because SBI has been implemented only in level I trauma centers, its impact on nationwide levels of recidivism may have been insignificant. A question arises from this, would expanding the SBI mandate to EDs nationwide reduce rates of recidivism among injured populations? Clearly, further research is warranted to support this expansion. Alcohol use is third leading cause of preventable death, accounting for more than 75,000 deaths annually (CDC 2015). Therefore, there is a significant need for a continuous prevention effort to reduce alcohol’s impact on population health.

The possibility remains that although SBI may reduce alcohol consumption, it may have no effect on injury recidivism. Although previous studies have found some evidence of reduction in injury recidivism following SBI programs, those studies were conducted more than a decade before the mandate and were limited to a single health institution. Therefore, the generalizability of those findings remains uncertain.

These results have implications for public health policy and practice. First, the estimated nine million recidivists represents a reference point for future prevention strategies to measure progress in reducing injury recidivism rate nationwide. Second, the study highlights the role depression plays in injury recidivism. Because mental health is a rising concern in the U.S. with over 16 million suffering from depression, reducing associated risks of repeated injuries should be among priorities of injury prevention programs. (Tice 2014) Third, we identified potential target populations for preventions including males between 18 and 25 years of age, those with diabetes, stroke or asthma. Finally, our study highlighted geographical differences in recidivism as individuals residing in the western region of the country had a 21 % higher odds of recidivism than those in the northeast. This finding may support local programs in this region to establish prevention initiatives by engaging the public and policymakers.

This study has several limitations that need to be taken into account in light of these findings. For instance, the MEPS did not include a question on self-reported alcohol use and frequency. To address this, we used a biological measure of health conditions associated with alcohol abuse, which may not capture accurate levels of alcohol use. However, a previous study (Rivara et al. 1993) found biochemical measures, such as blood alcohol concentration, to be significant predictors of recidivism while behavioral measures were not in the same population. Another important limitation in this study was the omission of injury mechanism due to the fact that MEPS stopped collecting mechanism data in panel 10. Previous studies identified violent injuries as one of the major risk factors for injury recidivism (Rowhani-Rahbar et al. 2015). Future research should take this into account in order to learn more about opportunities for prevention.

Despite limitations, this study is the first attempt to examine the rates of injury recidivism in the U.S. Unlike prior studies, we used a nationally representative sample and followed participants for over two years. We also take a more comprehensive approach to capture cases by looking at both all injured individuals whom their health was affected regardless of healthcare utilization and at those with severe injuries who sought medical care.

## Conclusion

In conclusion, we observed a higher recidivism rate among injured individuals in this study than previously reported. These nationally representative findings emphasize the pressing need for further prevention initiatives to reduce the burden of repeated injuries. Preventative efforts aimed to reduce injury recidivism may benefit from focusing on males between 18 and 25 years of age and those with depressive symptoms, diabetes, asthma and strokes.

## Abbreviations

AHRQ: Agency for Healthcare Research and Quality
ED: emergency department
MEPS: Medical Expenditure Panel Survey
SBI: screening and brief intervention
